# Birthweight DNA methylation signatures in infant saliva

**DOI:** 10.1186/s13148-021-01053-1

**Published:** 2021-03-19

**Authors:** Chiara Moccia, Maja Popovic, Elena Isaevska, Valentina Fiano, Morena Trevisan, Franca Rusconi, Silvia Polidoro, Lorenzo Richiardi

**Affiliations:** 1 Cancer Epidemiology Unit, Department of Medical Sciences, University of Turin and CPO Piemonte, Via Santena 7, 10126 Turin, Italy; 2 Unit of Epidemiology, ‘Anna Meyer’ Children’s University Hospital, Florence, Italy; 3 Italian Institute for Genomic Medicine (IIGM), Candiolo, Italy; 4 MRC-PHE Centre for Environment and Health, School of Public Health, Imperial College, London, UK

**Keywords:** Saliva DNA methylation, Birthweight, Infants, Birth cohort

## Abstract

**Background:**

Low birthweight has been repeatedly associated with long-term adverse health outcomes and many non-communicable diseases. Our aim was to look-up cord blood birthweight-associated CpG sites identified by the PACE Consortium in infant saliva, and to explore saliva-specific DNA methylation signatures of birthweight.

**Methods:**

DNA methylation was assessed using Infinium HumanMethylation450K array in 135 saliva samples collected from children of the NINFEA birth cohort at an average age of 10.8 (range 7–17) months. The association analyses between birthweight and DNA methylation variations were carried out using robust linear regression models both in the exploratory EWAS analyses and in the look-up of the PACE findings in infant saliva.

**Results:**

None of the cord blood birthweight-associated CpGs identified by the PACE Consortium was associated with birthweight when analysed in infant saliva. In saliva EWAS analyses, considering a false discovery rate *p*-values < 0.05, birthweight as continuous variable was associated with DNA methylation in 44 CpG sites; being born small for gestational age (SGA, lower 10^th^ percentile of birthweight for gestational age according to WHO reference charts) was associated with DNA methylation in 44 CpGs, with only one overlapping CpG between the two analyses. Despite no overlap with PACE results at the CpG level, two of the top saliva birthweight CpGs mapped at genes associated with birthweight with the same direction of the effect also in the PACE Consortium (*MACROD1* and *RPTOR*).

**Conclusion:**

Our study provides an indication of the birthweight and SGA epigenetic salivary signatures in children around 10 months of age. DNA methylation signatures in cord blood may not be comparable with saliva DNA methylation signatures at about 10 months of age, suggesting that the birthweight epigenetic marks are likely time and tissue specific.

**Supplementary Information:**

The online version contains supplementary material available at 10.1186/s13148-021-01053-1.

## Background

The existence of a relationship between intrauterine or early life exposures and health during the lifecourse has come to attention in the 1990s [1] and is nowadays recognized as the developmental origins of health and diseases (DOHAD) hypothesis [2]. The intrauterine life is a critical period for adverse exposures to exert their effect [3], as fetal organs start developing and are sensitive to environmental stimuli that may cause an indelible imprint on future development and function.

In a hostile uterine environment caused by insults, for example poor nutrition, the fetus, to slow down its growth rate in order to match the nutrient supply, responds by developing adaptations including down-regulation of metabolic or organs function. The adaptive process, however, may cause irreversible changes in the development of some tissues and organs and predispose an individual to a higher risk of diseases not only early in life, but during the entire lifecourse [2].

Low birthweight may be associated with accumulation of adipose tissue and rapid weight gain during childhood [3], and the risk of respiratory [4, 5], metabolic [6, 7] and cardiovascular diseases [8, 9], hypertension [10], and neurobehavioral disorders [11]. Low birthweight has also been associated with an increased overall mortality [12], while cancer incidence rises with increasing birthweight for most types of cancer [13–17].

The Pregnancy and Childhood Epigenetics (PACE) Consortium conducted so far the largest cord blood epigenome-wide DNA methylation study of birthweight using 8825 neonatal blood samples from 24 birth cohorts [18] and found that 914 CpGs, located in or near 729 genes, were associated with birthweight treated as a continuous variable. In the same study, methylation variation in 51 CpG sites was associated with high birthweight, as compared to normal birthweight, and 4 CpGs appeared to be associated with low versus normal birthweight. In additional analyses conducted on blood from 7278 children at later ages, only 1.3% of the 914 birthweight-associated differentially methylated CpGs in cord blood remained associated in childhood (2–13 years; *n* = 2756 children from ten studies), one in adolescence (16–18 years; *n* = 2906 from six studies), and none in adulthood (30–45 years; *n* = 1616 from three studies).

Although it seems that cord blood DNA methylation markers of birthweight do not persist at later ages, the associations observed at birth are extensive and it is important to confirm their persistence or variations over time. Also, DNA methylation profiles are tissue specific, and it would be optimal to analyse the DNA profile linked to birthweight in different tissues. Most tissues are not accessible with non-invasive methods, and blood is typically used as a surrogate, with the assumption that, being a universal body fluid, it may capture epigenetic changes of target tissues [19]. However, blood samples collection may be difficult in large population studies, as parents may be less prone to expose their children, and especially infants, to vein puncture for blood donation. Saliva and nasal brushes are easily accessible tissues, but to date they have been much less studied [20, 21]. Replication in saliva of blood epigenetic signatures of exposures and/or outcomes and identification of saliva-specific methylation markers could make saliva an optimal candidate for studies in infants and children. Thanks to its non-invasive collection method, it would allow obtaining repeated samples in short time periods, which is necessary requisite for monitoring changes in DNA methylation over time.

Our aim was to perform a look-up, in infant saliva samples, of birthweight-related DNA methylation variation identified in cord blood, and to conduct, to our knowledge, the first saliva epigenome-wide association study (EWAS) of birthweight, to identify methylation markers that may be specific for saliva collected in infancy.

## Methods

### Study population

The data were derived from an epigenome-wide case–control study on wheezing nested within the NINFEA birth cohort [22]. The NINFEA study is an Italian web-based multi-purpose mother–child cohort, aimed at exploring the relationship between early-life exposures and long-term health outcomes [23]. Members of the cohort are children born from approximately 7500 pregnant women who between 2005 and 2016 volunteered to participate in the study, had Internet access, and had enough knowledge of Italian to complete web-based questionnaires. The children are followed up with six questionnaires completed by their mothers 6 and 18 months after delivery, and when they turn 4, 7, 10, and 13 years of age. When children were aged approximately 6 months, mothers were asked to donate their and their children’s saliva samples using a mailed sponge Oragene™ DNA self‐collection kits (OG‐250; DNA Genotek, Inc, Ottawa, Ontario, Canada). Approximately half of the participating mothers donated their and their child saliva samples, which are stored in a biobank at −80 ºC.

The case–control study was designed as EWAS of early childhood wheezing, consisting of 72 cases with at least one episode of wheezing between 6 and 18 months of age, and 72 infants without wheezing matched to cases by sex, age, and seasonality/calendar year of saliva sampling. Cases and controls were selected from a subpopulation of singletons, residents in the City of Turin, Italy, and born to mothers who did not report having asthma active during pregnancy. The baseline NINFEA questionnaire, completed at any time during pregnancy, was used to derive information on maternal and pregnancy factors, while child-related variables were collected at the first follow-up questionnaire completed approximately 6 months after delivery. Although information on children ethnic background was not available in the NINFEA cohort, almost the entire study population has both parents born in Italy, and only few study children have one of the parents born in other European countries. Therefore, the ethnic background of the children included in the study is, if not entirely, largely European. In this study, we used the following variables: maternal age at delivery (years), maternal education (low = no education, primary or secondary school vs. high = university degree or higher), parity (nulliparous vs. at least one previous pregnancy > 22 gestational weeks), maternal pre-pregnancy body mass index (BMI; kg/m^2^), sex, gestational age at birth (weeks), birthweight (grams), small for gestational age (yes vs. no), age at saliva sampling (continuous in months) (see Table 1 for detail).

**Table 1:** Descriptive table for the NINFEA population under study (*N* = 141)

Characteristics	*N* or mean	% or SD
Maternal pre-pregnancy BMI^a^	22	3.0
Missing values	1	–
Maternal age at delivery (years)	35	4.1
Maternal education		
Low/medium (no university degree)	38	27%
High (university degree or higher)	102	72%
Missing values	1	1%
Parity		
First born	45	32%
Non first born	92	65%
Missing values	4	3%
Gestational duration (weeks)		
< 37 weeks	7	5%
37+ weeks	134	95%
Child sex		
Female	61	43%
Male	80	57%
Birthweight (grams)	3242	447.1
Low (< 2500 g)	5	3.5%
Medium (2500–4000 g)	128	90.8%
High (> 4000 g)	8	5.7%
Small-for-gestational-age^b^		
Yes	22	16%
No	119	84%
Age at saliva sampling (months)	10.78	2.2

^a^BMI: body mass index (Kg/m^2^)

^b^Based on the 10th percentile of the WHO growth reference charts

The Illumina Infinium ® HumanMethylation450 BeadChip (Illumina,Inc, San Diego, CA, USA) was employed to evaluate DNA methylation status of over 485,000 probes in saliva samples. Details on pre‐processing of samples and data quality control can be found in the Additional File 1 (Methods, DNA methylation measurement, data pre-processing, and quality control). Quality controls and probes filtering led to the exclusion of three samples and 63,218 probes, leading to a total of 141 saliva samples and 421,782 probes for analyses.

The NINFEA study was approved by Ethical Committee of the San Giovanni Battista and CTO/CRF/Maria Adelaide Hospital of Turin, and all participating mothers gave informed consent at enrolment and at saliva donation.

### Statistical analyses

For all the analyses, we pooled together cases and controls, leading to a total of 141 subjects. The analyses were, however, based on 135 subjects, as 6 (4.3%) infants had missing values in at least one of the variables included in multivariable models. Methylation levels were analysed as β-values (ratio of methylated probe intensity to overall intensity, representing 0 to 100% methylation at each probe). Although, for normality assumption, log 2-transformed β-values (M-values) may perform better when DNA methylation is used as an outcome, the interpretation of coefficients may be less intuitive. We, therefore, used β-values for more intuitive biological interpretation and easier comparability with other studies, in particular with the PACE birthweight EWAS. All the analyses were performed using R statistical computing software (version 3.6.0) and RStudio (version 1.2.1335) [24].

#### Look-up of PACE findings in infant saliva

Out of the 914 birthweight-associated CpGs identified by the PACE Consortium, 891 (97.5%) were available in the NINFEA study after quality checks and probes filtering. We used the same confounding variables of the PACE analysis, but, differently than in the PACE study, we used birthweight as the exposure and DNA methylation variation as the outcome to meet the biological temporality from birth to infancy. Both birthweight and DNA methylation were modeled as continuous variables. We used robust linear regression models adjusted for maternal age at delivery, maternal education, parity, maternal pre-pregnancy BMI, child sex, gestational age at birth, age at saliva sampling, batch, and case–control status of the original nested case–control study (wheezing between 6 and 18 months of age). Using *vcovHC* function in the sandwich R package [25], we calculated heteroscedasticity-consistent standard errors.

Although maternal smoking during pregnancy affects both birthweight [26] and offspring DNA methylation [27], we did not adjust for smoking, as it was rather infrequent in our study sample (2% prevalence). In order to account for residual technical variability and for cell-type heterogeneity, we performed surrogate variables analysis (*sva*) [28] and estimated 7 surrogate variables that were also included as covariates in the model. Cell composition was additionally estimated using the reference-based projections for saliva proposed by Zheng [29], and given a high correlation between the epithelial tissue component estimate and the first sva component (rho = 0.99), we only used the 7 estimated surrogate variables in the analyses. *p*-Values adjusted for multiple comparisons were calculated using the Bonferroni correction and the Benjamini and Hochberg false discovery rate (FDR), while histograms and quantile–quantile (QQ) plots were used to graphically compare the observed distribution of *p*-values versus the expected uniform distribution under the null hypothesis. Given that the direction of the association was determined by the PACE study, we also calculated one sided *p*-values for each CpG. It has been shown that CpGs associated with a certain trait tend to be highly correlated and that this correlation affects standard procedures for multiple testing, such as Bonferroni and Benjamini and Hochberg corrections. In the NINFEA saliva samples, the correlation between the 891 CpGs identified in the PACE EWAS was 0.48, which is indeed much higher than the reported saliva genome-wide mean correlation of 0.12 [30]. We, therefore, reported the permutation *p*-values that take into account the distribution of *p*-values under the null hypothesis and are suggested as a gold standard for the settings where the underlying correlation between CpGs is high.

#### Epigenome-wide association analyses

Two epigenome-wide association analyses were conducted, first with birthweight as a continuous exposure variable and, second, with small for gestational age (SGA) as a binary exposure variable. The latter was defined as the lowest 10^th^ percentile of the World Health Organization birthweight for sex and gestational age charts [31], with the remaining population above the 10th percentile as the reference group. As in our study population only 7 children (5%) were classified as large for gestational age (based on the 90^th^ percentile), we had no power to analyse this trait separately and opted for a more conservative approach by including them in the reference group. In both EWAS analyses, we used robust linear regression models adjusted for sex, age at saliva sampling, gestational age, maternal age, parity, maternal pre-pregnancy BMI, maternal education, batch, estimated surrogate variables, and case–control status of the original nested case–control study. *p*-Values adjusted for multiple comparisons were calculated using the Benjamini and Hochberg false discovery rate (FDR), and Volcano plots were used to visually present the results. In the EWAS of SGA, as a sensitivity analysis we provisionally excluded children born pre-term (< 37 gestational weeks at birth).

To assess whether the age at saliva sampling could have influenced the findings on the top CpG sites identified in the EWAS analyses, we tested the associations of the age at saliva sampling as a continuous variable (in months) with the methylation levels in the top CpG sites using the robust linear regression models adjusted for sex, batch and cell type composition estimated with the reference-based projections for saliva proposed by Zheng [29].

Finally, we performed a look-up of the saliva top findings in the PACE summary results, publicly available via Zenodo: https://zenodo.org/record/2222287#.YDOhCtWSmUk.

### CpGs annotation and functional analysis

Gene Ontology (GO) and Kyoto Encyclopedia of gene and Genomes (KEGG) enrichment analyses were carried out to identify possible functional pathways in the saliva birthweight-associated CpGs set.

In order to compare previously reported associations of epigenome-wide birthweight-associated CpGs and our own results, we searched for findings reported in the EWAS Catalog (http://www.ewascatalog.org; accessed on 12 February 2021) and EWAS Atlas (https://bigd.big.ac.cn/ewas/tools; accessed on 12 February 2021), looking for both CpG and gene level match.

After this first step based on the overlap between our results and those of other EWAS on the same trait, we further accessed EWAS Atlas to examine whether CpGs identified in our study were previously associated with traits other than birthweight.

We also looked in the GWAS Catalog for traits associated with specific single-nucleotide polymorphisms (SNPS) on genes on which these CpGs mapped.

## Results

Table 1 shows the characteristics of the study population. The mean maternal age was 35 years; 72% of the mothers had a high educational level. The mean age at saliva sampling was 10.8 months (median 10.4, range 7–17). The mean birthweight was 3242 g, 5% of the children were born pre-term and 15.6% of children were born small for gestational age, respectively).

### Look-up of PACE findings in infant saliva

**Fig. 1:**
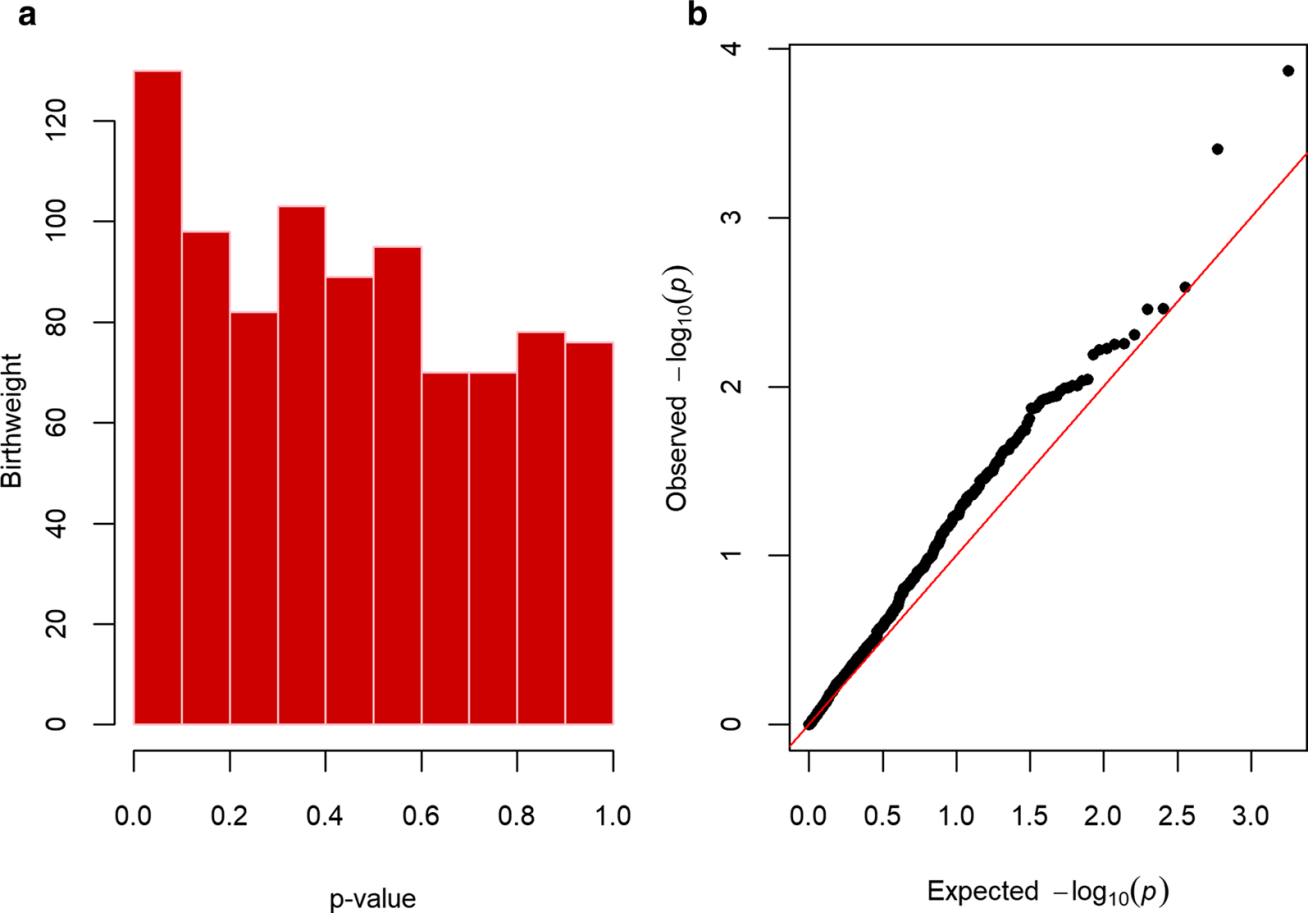
Histogram and qq-plot of the two-sided *p*-values from the look-up of PACE cord blood findings in infant saliva

Of the 891 CpG sites associated with birthweight in the PACE study, 52 (5.8%) had a one sided *p*-value < 0.05 in our study (Fig. 1, Table 2). However, none of the CpGs survived the Bonferroni correction (*p* < 5.61 × 10^−5^) or had a FDR < 0.05 (Table 2, Additional file 2: Table S1). There was a 47% concordance in the direction of the coefficients between the PACE and our study (binomial sign test *p*-value = 0.11, 95% confidence intervals (CIs) 0.44; 0.51). When we used the permutation test to take into account strong mean pairwise correlation between the DNA methylation values of the 891 CpG sites, the minimum permutation *p*-value was 0.56.

**Table 2:** Results from the look-up of the PACE cord blood findings in the NINFEA saliva samples

Results	*N* (%)
Number of CpGs tested	891
Number of CpGs with two sided *p*-value < 0.05	81 (9.1%)
Number of CpGs with one sided *p*-value < 0.05	52 (5.8%)
Number of CpGs concordant in the direction of the effect between the PACE and NINFEA study	421 (47.3%)
Family-wise error rate (Bonferroni-corrected *p*-values) < 5.61 × 10^−5^	0
Number of CpGs with permutation-corrected *p*-value < 0.05	0

### EWAS for continuous birthweight

**Fig. 2:**
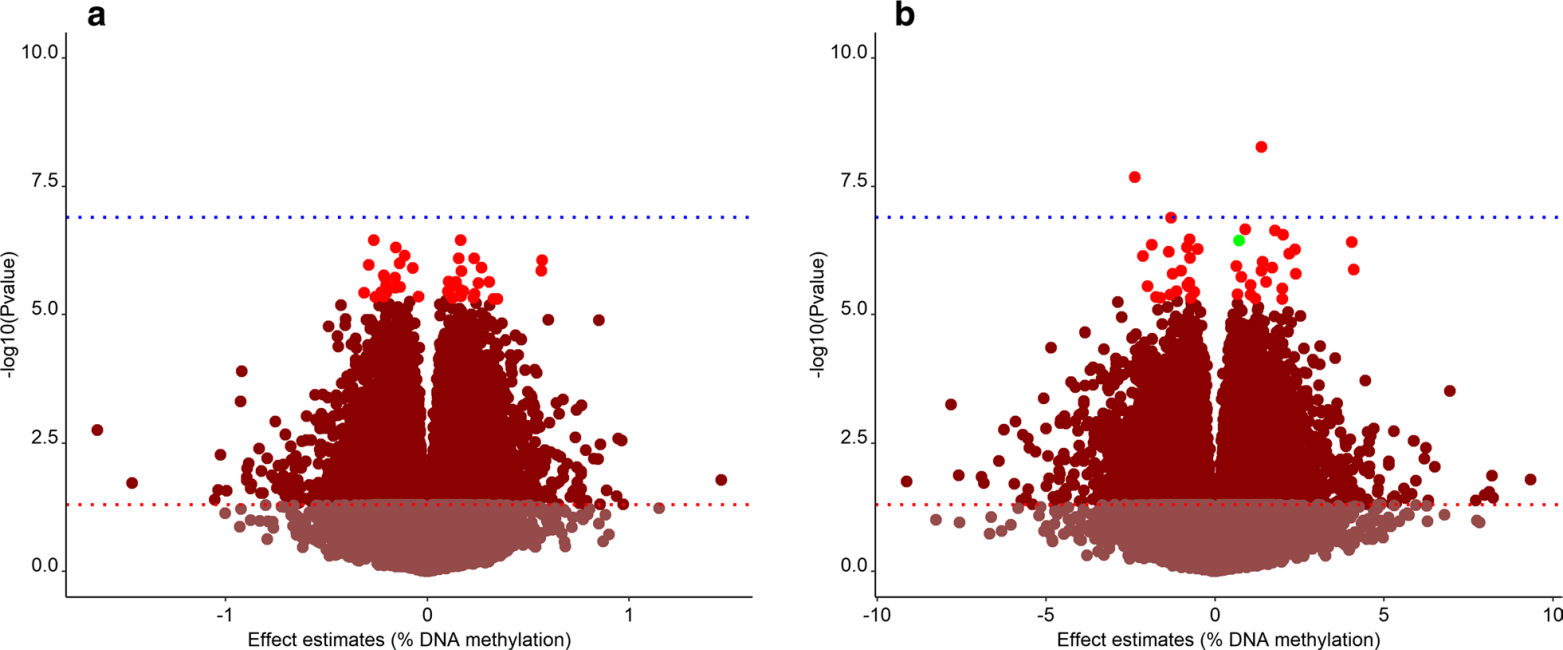
Volcano plot of the two EWAS. **a** Volcano plot showing *p*-values and direction of associations of DNA methylation variation with continuous birthweight. 44 FDR < 0.05 highlighted in red. The blue line is Bonferroni threshold, the red line nominal *p*-value threshold (*p* < 0.05). The *X*-axis represents the % difference in methylation per 100 g in birthweight difference, and the *Y*-axis represents the − log10(*p*-value). **b** Volcano plot showing *p*-values and direction of associations of DNA methylation variation with AGA vs. SGA. 44 FDR < 0.05 highlighted in red. The blue line is Bonferroni threshold, the red line nominal *p*-value threshold (*p* < 0.05).The *X*-axis represents the % difference in methylation per AGA vs. SGA, and the *Y*-axis represents the − log10(*p*-value). In green the CpG that overlap with 44 CpGs on continuous birthweight

Out of the 421,782 probes analysed, 8.9% (*N* = 37,365) were associated with birthweight with a nominal *p*-value < 0.05. After correction for multiple testing, 44 CpG sites had a FDR < 0.05 (Table 3 and Fig. 2a). Their coefficient estimates ranged from – 0.31 to 0.57, which corresponds to a methylation increase of 0.57% with a 100 gr increase in birthweight. The largest effect was observed for cg02727104 located 13 kb down *SOHLH2*.

**Table 3:** Top 44 CpGs from EWAS study with Benjamini and Hochberg false discovery rate (FDR)‐adjusted *p*‐values < 0.05. The effect is estimate as the difference in % of methylation per 100 g in birthweight difference. In bold the CpG with the strongest effect

CpG	Chromosome	Gene allocation or nearest gene	Effect estimate	Standard error	Nominal *p*-value	FDR corrected *p*-value
cg03045325	11	*MACROD1*	− 0.266	4.89E−06	3.53E−07	0.046
cg19854704	10	95 kb down *ENSG00000286295*	0.165	3.03E−06	3.56E−07	0.046
cg07728793	2	*RAMP1*	− 0.155	2.90E−06	4.91E−07	0.046
cg18072629	10	*GATA3*	− 0.112	2.13E−06	7.03E−07	0.046
cg05005073	1	10 kb up *NDUFS5*	0.233	4.44E−06	8.01E−07	0.046
cg09516627	2	*CENPO*	0.155	2.97E−06	8.07E−07	0.046
**cg02727104**	**13**	**13 kb down SOHLH2**	**0.569**	**1.09E−05**	**8.7E−07**	**0.046**
cg26725813	15	*ZNF770*	− 0.136	2.63E−06	9.99E−07	0.046
cg05931551	1	*TUT4*	− 0.290	5.61E−06	1.08E−06	0.046
cg26392737	8	*DMTN*	0.268	5.21E−06	1.21E−06	0.046
cg04512603	7	*ZNF273*	− 0.072	1.39E−06	1.24E−06	0.046
cg23218354	1	50 kb down *ACTRT2*	0.565	1.10E−05	1.4E−06	0.046
cg09855212	11	*SYTL2*	0.169	3.32E−06	1.43E−06	0.046
cg04305601	16	*ZNF423*	− 0.215	4.25E−06	1.72E−06	0.046
cg13590166	10	70 kb down *WAC*	− 0.211	4.18E−06	1.86E−06	0.046
cg01807862	5	*MCIDAS*	− 0.159	3.16E−06	1.91E−06	0.046
cg14781041	1	*SIPA1L2*	0.105	2.10E−06	2.25E−06	0.046
cg15915658	11	30 kb down *SCYL1*	0.307	6.14E−06	2.3E−06	0.046
cg03466415	2	*SCN9A*	0.142	2.85E−06	2.32E−06	0.046
cg22453818	5	*LHFPL2*	0.254	5.09E−06	2.43E−06	0.046
cg07175848	16	*SNTB2*	− 0.204	4.11E−06	2.7E−06	0.046
cg18971416	10	*THNSL1*	0.129	2.60E−06	2.72E−06	0.046
cg18152712	6	*E2F3*	0.115	2.32 E−06	2.76E−06	0.046
cg08060902	19	ZNF709	− 0.151	3.05 E−06	2.8E−06	0.046
cg04963607	7	*PRKAR1B*	− 0.135	2.72 E−06	2.87E−06	0.046
cg00483825	10	*CFAP46*	− 0.157	3.19 E−06	2.99E−06	0.046
cg18417562	5	*CTNNA1*	− 0.198	4.03 E−06	3.07E−06	0.046
cg09361653	17	*RPTOR*	− 0.192	3.91 E−06	3.18E−06	0.046
cg22896429	6	*MTHFD1L*	0.165	3.37 E−06	3.33E−06	0.046
cg19794939	11	*LGR4*	0.101	2.07 E−06	3.48E−06	0.046
cg03113121	19	*FOXA3*	0.178	3.63 E−06	3.49E−06	0.046
cg23114964	1	*HIVEP3*	− 0.229	4.69 E−06	3.67E−06	0.046
cg06527318	1	*MIR4425*	− 0.313	6.42 E−06	3.77E−06	0.046
cg20100049	11	*KMT5B*	− 0.204	4.18 E−06	3.84E−06	0.046
cg20515787	11	SART1	0.108	2.21 E−06	3.91E−06	0.046
cg13680864	10	STAM	0.235	4.82 E−06	3.96E−06	0.046
cg23096644	7	SGCE	0.167	3.44 E−06	4.37E−06	0.047
cg03722643	2	*GPR45*	− 0.044	9.07 E−07	4.43E−06	0.047
cg19857227	14	*ENSG00000240914*	− 0.215	4.45 E−06	4.49E−06	0.047
cg08707988	6	*41 kb up LOC100294145*	− 0.258	5.33 E−06	4.51E−06	0.047
cg09749788	8	*MCPH1-AS1*	0.229	4.76 E−06	4.7E−06	0.047
cg06655187	17	*ZNHIT3*	0.125	2.59 E−06	4.79E−06	0.047
cg10800369	12	*LMO3*	0.327	6.80 E−06	4.91E−06	0.047
cg24864887	15	*800b down ISL2*	0.347	7.20 E−06	4.92E−06	0.047

None of the 44 saliva birthweight-associated CpGs overlapped with the birthweight related CpGs identified in cord blood in the PACE study under the Bonferroni threshold. When considering a nominal *p*-value < 0.10 in the PACE study (*N* = 78,489 CpGs), there was an overlap in 6 CpG sites, but only one with the same direction of the effect (cg22896429) (Additional file 3: Table S1). At gene level, there was an overlap in 12 genes identified in PACE under the FDR-corrected *p*-value threshold (with at least one CpG with the same direction of the effect). The average absolute pairwise Pearson's correlation coefficient between the β-values of the identified 44 CpGs was 0.23, which is higher than the mean pairwise genome-wide correlation coefficient in the NINFEA saliva samples but lower than the mean pairwise correlation coefficient between 891 CpGs identified by the PACE Consortium [30].

### EWAS for small for gestational age

We found 44 CpGs associated with SGA at a FDR of less than 0.05 (Table 4 and Fig. 2b). The largest coefficient showed a 4.1% difference in methylation when comparing small with non-small for gestational age (cg12322146, located 125 kb down *RBMS3*); the largest negative association was -2.3% (cg03066788, located at *PNOC*). Only one CpG (cg18072629, located at *GATA3*) overlapped with the 44 CpGs found to be associated with continuous birthweight in our sample. None of the 44 saliva SGA-associated CpGs overlapped with the 914 CpGs associated with continuous birthweight (at Bonferroni threshold), or with the 4 CpGs associated with low birthweight (< 2500 g) in the PACE study conducted on cord blood samples. When considering a nominal *p*-value < 0.10 in the PACE study, there was an overlap in 7 CpG sites, but only two with consistent direction of the effect (cg06234201, cg15847996). (Additional file 3: Table S1). At gene level, there was an overlap in 9 genes identified in PACE under the FDR-corrected *p*-value threshold (with at least one CpG with consistent direction of the effect).

**Table 4:** 44 CpGs associated with AGA vs. SGA when treats birthweight as categorical variable. The coefficient estimates are the difference in % of methylation per AGA vs. SGA. In bold the CpG with the strongest effect

CpG	Chromosome	Gene allocation or nearest gene	Effect estimate	Standard error	Nominal *p*-value	FDR corrected *p*-value
cg26168577	17	*MRC2*	1.374	2.17E−03	5.36E−09	0.002
cg03066788	8	*PNOC*	− 2.371	3.92E−03	2.07E−08	0.004
cg26615232	1	*USH2A*	− 1.304	2.31E−03	1.3E−07	0.017
cg12055114	19	*LMNB2*	0.898	1.62E−03	2.18E−07	0.017
cg04873627	4	*TBC1D19*	1.771	3.20E−03	2.3E−07	0.017
cg15225594	5	20 kb down *ERCC8*	2.016	3.68E−03	2.76E−07	0.017
cg01286950	2	*AFF3*	− 0.747	1.37E−03	3.4E−07	0.017
cg18072629	10	*GATA3-AS1*	0.715	1.32E−03	3.62E−07	0.017
cg13984701	9	*COL5A1*	4.051	7.49E−03	3.88E−07	0.017
cg26332310	1	*BARHL2*	− 1.867	3.47E−03	4.39E−07	0.017
cg24391471	3	*ZDHHC3*	− 0.829	1.55E−03	4.84E−07	0.017
cg02547025	2	*LBH*	− 0.517	9.69E−03	5.26E−07	0.017
cg23508813	3	2.5 kb down *LOC100507274*	2.372	4.45E−03	5.33E−07	0.017
cg26589351	17	*SPOP*	− 1.370	2.58E−03	5.97E−07	0.018
cg06234201	16	*DUS2*	2.202	4.16E−03	6.49E−07	0.018
cg23954819	12	*BRI3BP*	− 2.136	4.06E−03	7.26E−07	0.019
cg19445996	17	*RAB37*	− 0.744	1.42E−03	7.85E−07	0.019
cg02980127	14	14,5 kb down *ENSG00000258736.1*	1.402	2.70E−03	9.4E−07	0.022
cg22869025	20	*ACSS1*	0.629	1.22E−03	1.14E−06	0.025
cg19403534	10	*PRLHR*	1.688	3.28E−03	1.22E−06	0.025
**cg12322146**	**3**	**125 kb down RBMS3**	**4.106**	**8.01E−03**	**1.31E−06**	**0.025**
cg06458489	5	150 kb down *SNORA63*	− 0.999	1.95E−03	1.38E−06	0.025
cg00383136	6	*HLA-DRA*	1.370	2.68E−03	1.39E−06	0.025
cg16899265	18	*GRP*	− 1.257	2.47E−03	1.59E−06	0.027
cg15847996	10	*INPP5A*	2.390	4.71E−03	1.61E−06	0.027
cg08715720	5	*PPP2R2B*	0.780	1.55E−03	1.83E−06	0.030
cg20388707	2	*NGEF*	1.510	3.03E−03	2.31E−06	0.036
cg01504555	16	*SYCE1L*	− 0.766	1.54E−03	2.4E−06	0.036
cg07965823	14	*ISM2*	− 0.820	1.65E−03	2.64E−06	0.036
cg20244295	15	*ZNF710*	1.049	2.12E−03	2.65E−06	0.036
cg09311778	17	2 kb down *KAT7*	− 0.795	1.61E−03	2.78E−06	0.036
cg05754929	16	*GSE1*	− 1.993	4.03E−03	2.81E−06	0.036
cg18246134	3	*LINC02877*	− 0.767	1.55E−03	2.85E−06	0.036
cg21688288	21	*PDE9A*	1.994	4.05E−03	3.08E−06	0.038
cg22159939	1	30 kb down *CENPF*	− 1.137	2.32E−03	3.49E−06	0.042
cg25271404	15	*GOLGA8B*	− 0.617	1.26E−03	3.62E−06	0.042
cg24745895	7	5 kb up *AKR1D1*	0.669	1.38E−03	4.01E−06	0.044
cg17836487	2	*TMEM131*	− 1.328	2.73E−03	4.07E−06	0.044
cg24113784	17	*MIEF2*	1.050	2.16E−03	4.08E−06	0.044
cg00701706	17	*OTOP3*	− 1.747	3.62E−03	4.57E−06	0.048
cg24663455	10	50,5 kb down *MGMT*	− 1.615	3.35E−03	4.73E−06	0.048
cg08935613	11	*HYLS1*	− 0.720	1.50E−03	4.83E−06	0.048
cg22213242	11	*CD248*	1.191	2.48E−03	4.91E−06	0.048
cg24420742	12	*NAV3*	1.994	4.15E−03	4.96E−06	0.048

Findings were practically unchanged when we excluded preterm infants from the EWAS analysis (data not shown). We found no association between age at saliva sampling and methylation status for any of the top CpGs associated with continuous birthweight or with SGA in our two EWAS analyses.

Out of 44 CpG sites associated with continuous birthweight in our EWAS, 28 (64%) showed a nominal *p*-value < 0.05 in the EWAS for SGA; all with a direction of the effect that was consistent with the results for birthweight. Likewise, 26 (59%) out of 44 SGA-related CpGs reveal nominal *p*-value < 0.05 in the EWAS of continuous birthweight, with a consistent direction of the effect.

### CpGs Annotation and functional analysis

None of the two sets of CpGs identified in our study, the 44 birthweight-associated CpGs and the 44 SGA-associated CpGs, showed functional enrichment of GO or KEGG terms.

Also, there was no overlap between the CpGs identified in our EWAS on continuous birthweight and 995 birthweight-related CpGs reported in 4 cord-blood and subcutaneous adipose tissue studies from the EWAS Atlas data [32–34], including the PACE study [18]. In EWAS Atlas and EWAS Catalog, DNA methylation in six out of 87 genes was previously associated with birthweight, all in the PACE study at Bonferroni threshold. In addition, some genetic variants in these six genes have been previously associated with obesity [35], adult BMI [36–42], BMI-adjusted waist-hip ratio [37, 38, 41, 43, 44], high-density lipoprotein cholesterol measurement [45–47], and visceral adipose tissue measurement [48].

In the EWAS Catalog, there were 34 CpGs associated with birthweight in four studies using DNA methylation in cord blood [49–52], but none of these overlapped with the 87 CpGs found to be associated with birthweight or SGA in our study. In the EWAS Atlas, DNA methylation in 29 out of these 87 CpGs was associated with different traits (Additional file 1: Tables S1 and S2). DNA methylation variation at eleven of them has been previously associated with insulin resistance [53] (cg03045325), colorectal cancer [54] (cg02727104, cg26332310, cg12322146), obesity [55] (cg03066788), bariatric surgery [56] (cg20515787, cg02547025), mortality [57] (cg06234201), gestational diabetes mellitus [58] (cg00383136), amount of visceral adipose tissue [59] (cg20388707), and gestational age [34] (cg00701706).

Some of the birthweight- and SGA-associated CpGs in our study map in genes which variants were associated with the following traits: birthweight (*USH2A*), BMI (*CENPO, E2F3, RPTOR, SNTB2, PNOC, LGR4*), body fat distribution (*CENPO*), waist-hip ratio (*SYTL2, ZNF423, FOXA3, LMNB2, COL5A1,LGR4*), BMI-adjusted waist hip ratio (*ZNF423, AFF3*), cardiovascular disease (*SIPA1L2, FOXA3, RAB37, INPP5A*), subcutaneous or visceral adipose tissue measurement (*FOXA3, RPTOR*), gestational age (*CFAP46, INPP5A*), lipoprotein cholesterol measurement (*DMTN, SNTB2*), total cholesterol measurement (*E2F3, DMTN*), type I diabetes nephropathy (*AFF3*), type II diabetes mellitus (*RAMP1, SYCE1L, ZNF710*). Finally at gene level, out of saliva 87 CpGs associated with birthweight and SGA, DNA methylation at 14 genes was associated with birthweight, 22 with BMI and 32 with obesity in previous studies found in EWAS Atlas and EWAS Catalog. Full results are shown in Additional file 1: Tables S1 and S2.

## Discussion

In this study, we investigated the association between birthweight and methylation patterns in saliva samples taken at around 10 months of age. The cord blood methylation signatures of birthweight, found by a large study of the PACE Consortium, were not confirmed in infant saliva.

We identified 87 infant saliva-specific signatures of birthweight or SGA, of which two overlap with PACE results at gene level with the same direction of effect (*MACROD1* and *RPTOR)*. Interestingly, single-nucleotide polymorphisms in these genes have been previously associated with obesity, adult BMI, BMI-adjusted waist-hip ratio, high-density lipoprotein cholesterol and visceral adipose tissue levels. Moreover, expanding the look-up in the full PACE results, 13 CpGs find correspondence with a nominal *p*-value < 0.10 but only 3 have the same direction of the effect, namely cg22896429, cg06234201, cg15847996.

DNA methylation variation at some of the 87 loci identified in our study has been previously associated with multiple traits, such as insulin resistance, colorectal cancer, obesity, and gestational diabetes mellitus. Moreover, the SGA-associated locus cg26615232 maps within *USH2A,* which variant has been associated with birthweight in a study [63] that performed GWAS meta-analyses of fetal genetic variants in 321,223 individuals of European ancestry.

It has been repeatedly shown that DNA methylation at many sites is not temporally stable and that each tissue has its unique epigenetic landscape that likely reflects its specific function and response to environmental exposures [21, 64]. For example, a cross-sectional study on 1019 infants [65] compared the associations between preterm birth and genome-wide DNA methylation profiles using both cord tissue and cord blood samples. The results highlighted differences between the two tissues in DNA methylation variation associated with preterm birth, with only a minority of overlapping CpGs. In DNA from cord tissue, DNA methylation analysis showed enrichment of differentially methylated regions in genes involved in molecular pathways related to fetal growth and development (i.e. Wnt signaling, bone remodeling, and extracellular matrix organization), while in cord blood immune response pathways (i.e. regulation of T cell differentiation, inositol lipid-mediated signaling, and regulation of RNA stability) were enriched. Therefore, it is reasonable to speculate that saliva and blood, which have different functions, include different cell types and have different embryonic origin, and different mechanisms of exposure and responses to environmental factors do not share the same DNA methylation response to fetal growth, birthweight, and their risk factors. Although our results suggest different cord blood and saliva methylation patterns related to birthweight, we cannot exclude that the relatively small sample size of our study contributed to the lack of overlap with the PACE results, due to a reduced power to detect small effects.

In addition to the tissue specificity of DNA methylation, there are also age-related changes (birth vs. infancy) that could explain differences between our and the PACE Consortium findings.

In most tissues, DNA methylation may also vary substantially with time, especially during periods of life associated with high plasticity and fast development. Consistently, the PACE study found that differential methylation associated with birthweight in neonates persisted only minimally across childhood and disappeared by adulthood [18].

This result is consistent with another longitudinal study in which birthweight- and gestational age-related DNA methylation changes were investigated in cord blood and peripheral blood at ages 7 and 17 in more than 900 children [66]. Across the majority of CpG sites that showed differential methylation in cord blood, a pattern of fast evolution was observed during early childhood that stops with adolescence, providing evidence for the lack of persistence of early life methylation differences.

This is evident also in two studies that analysed infant saliva samples. A longitudinal study with repeated saliva samples collected at birth and at 1 year of age from 50 preterm and 40 infants born at term showed that DNA methylation at the differentially methylated region (DMR) of *IGF2* and *FKBP5* at birth was lower in preterm infants compared with term infants, but these differences did not persist at 1 year of age [67]. Also, another study on 214 infant saliva samples (62 collected at 6 weeks of age, 30 collected at 52 weeks of age and 61 collected at both ages) showed an age-dependent variation of DNA methylation, with a clear difference between saliva DNA methylation at 6 and 52 weeks of age [68].

In our study, we could not distinguish between the tissue- and the time-related differences in DNA methylation of birthweight-associated CpG sites, as we had no repeated saliva samples or blood samples collected simultaneously with saliva. It would be interesting in future studies with repeat samples and also with different tissues, to investigate whether the differences between our and PACE findings are due to the time- and tissue- dependent DNA methylation changes, as reported by previous studies both for birthweight and gestational age. Even though the age at saliva sampling varied between 7 and 17 months in our study, age was not associated with DNA methylation variation in any of the CpG sites associated with continuous birthweight and SGA, suggesting that the observed associations are unlikely influenced by the different age of saliva collection. It should be, however, noted that the age range of our population was rather narrow (7–17 months), so we cannot exclude that age could influence DNA methylation in these CpGs when considered across childhood/adolescence.

We supplemented the analysis of birthweight with the analysis on SGA and found different CpG sites associated with the two exposures, with only 1 overlapping CpG when considering FDR-corrected *p*-values. Although this is in line with the PACE analyses, where the 4 CpGs associated with low birthweight (< 2500 g) did not overlap with the 914 associated with continuous birthweight, approximately 60% of CpGs identified in our two EWAS overlapped at the nominal *p*-value of 0.05, with seemingly the same direction.

The main strength of our study was the possibility to analyse DNA methylation in saliva samples, which are easy to collect at any age in childhood and are, therefore, good candidates for future large DNA methylation studies. Moreover, while it is probably unfeasible to collect repeated blood samples over relatively short time periods, and especially in the context of large population studies based on children, it is possible to use saliva to monitor changes in DNA methylation over time. As salivary DNA methylation is poorly studied, especially in newborns and infants, it would be important to understand if, and for which specific traits and exposures, salivary and blood DNA methylation can be used interchangeably, and when they clearly show distinct methylation signatures. Previous DNA methylation studies using saliva samples focused on neurobehavioural conditions [69–71], respiratory traits [22], and cancer research [72], but to our knowledge the associations between birthweight and saliva DNA methylation has not been studied so far.

Although the small sample size of our study, especially in comparison with the PACE study, may have had an impact on EWAS analyses, after an FDR-correction, we identified some novel CpGs associated with birthweight, which are likely to be saliva-specific birthweight signatures. These findings need replication in independent saliva EWAS, and we cannot exclude that additional saliva DNA methylation variations related to offspring birthweight may be identified by larger studies. We argue that the sample size had less impact on our look-up analysis of PACE findings in saliva, as the number of tests performed in the look-up analysis was much lower than the number of tests of the two EWAS.

Our study population was not selected at random from the entire NINFEA cohort, but on the presence/absence of infant wheezing between 6 and 18 months of age, which was a selection factor for the original case–control study. As SGA is a well-known risk factors for wheezing [73, 74], this selection led to a relatively high prevalence of SGA infants (15.6%; 21% in wheezing cases and 10% in controls). To account for this selection, all the analyses were adjusted also for infant wheezing. Moreover, SGA definition is based on an arbitrary population-based reference cut-off, while, biologically, size for gestational age can be considered as a continuous trait. Therefore, the high prevalence of SGA in our study population allowed us to capture the lowest quintile of the size for gestational age distribution, providing more power for EWAS.

Finally, it should be noted that the prevalence of maternal smoking during pregnancy was rather low in our study population (2% compared with 7.7% of maternal smoking during pregnancy and with 14% of maternal smoking just before pregnancy in the entire cohort) [4]. In addition to random variation, the selection process may have played a role also in this case, although with an unexpected direction. Therefore, our findings are less generalizable to the population of women who smoke during pregnancy, but remain applicable to all non-smoking women and those who stopped smoking before pregnancy, which represent the majority of pregnant women.

## Conclusion

In conclusion, our study provides an indication of the birthweight and small for gestational age epigenetic salivary signatures in children around 10 months of age and suggests that DNA methylation signatures of birthweight likely differ between cord blood and infant saliva. Further insights are needed to understand whether these differences are due to biological differences between the two tissues or could be attributed to age-related DNA methylation changes.

## Supplementary Information

**Additional file 1**. DNA methylation measurement, data pre-processing, and quality control, traits associated in EWAS Atlas with 44-saliva birthweight related CpGs, traits associated in EWAS Atlas with saliva44-SGA-related CpGs.

**Additional file 2**. Results from the look-up of PACE findings.

**Additional file 3**. Look-up of the 87 saliva CpG sites in the PACE birthweight cord blood EWAS.

## Data Availability

Data from the NINFEA cohort underlying the findings reported in this study are available to researchers who meet the criteria for access to confidential data and upon reasonable request. Data availability contact: Prof. Lorenzo Richiardi (lorenzo.richiardi@unito.it).

## Abbreviations

CpG: Cytosine–guanine dinucleotide
AGA: Adequate for gestational age
SGA: Small for gestational age
FDR: False discovery rate
EWAS: Epigenome-wide association study
