# Supplementary material for: Birthweight DNA methylation signatures in infant saliva
Source: Clin Epigenetics. 2021 Mar 19;13:57. doi: 10.1186/s13148-021-01053-1 (PMC7980592; doi:10.1186/s13148-021-01053-1)
Supplement: Supplementary file 1 — Additional file 1. DNA methylation measurement, data pre-processing, and quality control, traits associated in EWAS Atlas with 44-saliva birthweight related CpGs, traits associated in EWAS Atlas with saliva44-SGA-related CpGs. [file 13148_2021_1053_MOESM1_ESM.docx]

*Birthweight DNA methylation signatures in infant saliva*

**Supplementary Material**

DNA methylation measurement, data pre-processing, and quality control………………………2

Supplementary S1Table …………………………………………………………………………..………………… 4

Supplementary S2 Table …………………………………………………………………………..………………….9

**Methods**

***DNA methylation measurement, data pre-processing, and quality control***

DNA was extracted from 144 saliva sponge samples using an automated protocol and the Oragene^TM^Purifier Solution (DNA Genotek, Inc., Ottawa, Ontario, Canada). Genomic DNA (500 ng) was bisulphite-converted using the EZ DNA Methylation-Gold^TM^ Kit (Zymo Research, CA, USA) following the manufacturer’s protocol.

The methylation status of over 485,000 probes was measured using the Infinium HumanMethylation450 BeadChip array (Illumina, Inc., San Diego, CA). The wheezing case-control pairs were matched on sex, season and age at saliva collection, and were placed on the same chip to minimize confounding by batch. Data pre-processing steps were performed in R statistical computing software version 3.4.1^1^ using the *minfi* package.^2^ DNA methylation for each probe was expressed as beta-value (ratio of methylated probe intensity to overall intensity, representing 0 to 100% methylation at each probe).

Three samples with more than 1% of the CpGs with detection p-value>0.01 were excluded from the study (N=3).To minimize the unwanted variation within and between samples the preprocessQuantile function was used.^3^ This function implements a stratified quantile normalisation procedure that is applied to the methylated and unmethylated signal intensities separately, and takes into account the different probe types.^3^ This procedure does not apply background correction, but very small intensities close to zero are thresholded using the *minfi* function fixMethOutliers.^2^

We filtered out probes that failed in one or more samples based on detection p-value (detection p-value>0.01; N=10,902), probes located on the sex chromosomes (N=10,831), all probes containing a single-nucleotide polymorphism at the CpG interrogation and/or at the single nucleotide extension for any minor allele frequency (using the *minfi* function dropLociWithSnps,^2^ N=15,654), and polymorphic CpG probes that have been demonstrated to map to multiple places in the genome^4^ (N= 26,343 probes). The final data set included 141 samples with DNA methylation measured in 421,782 probes.

S1Table. Traits associated in EWAS Atlas with 44-saliva birthweight related CpGs. The second column reports the methylation status of each locus in our analysis. In the sixth column are reported the traits found to be associated with each locus in the EWAS Atlas and the methylation status for each CpG in the EWAS Atlas studies (+=hypermethylated; -=hypomethylated; NR=not reported). In the 7th column are reported the traits found to be associated with genetic variants of the related nearest genes in the GWAS Catalog. In the last column are reported traits associated with DNA methylation at gene level.

| **CPG** | **Methy**  **lation**  **status** | **Chromo-some** | **Gene on which CpG is allocated or nearest gene** | **CpG location on the gene** | **Traits associated with DNA methylationin EWAS Atlas** | **Traits associated with gene variantsin GWAS catalog** | **Traits associated with DNA methylation at gene level in EWAS Atlas (1) and EWAS Catalog (2)** |
| --- | --- | --- | --- | --- | --- | --- | --- |
| cg03045325 | - | 11 | *MACROD1* | Body | insulin resistance NR |  | amount of visceral adipose tissue(1), birthweight (1)(2), BMI (1)(2), gestational diabetes mellitus(1), mortality (1)(2). obesity (1), type 2 diabetes, colorectal cancer(1), insuline resistance(1), bariatric surgery(1), Triglycerides to total lipids ratio in very large HDL(2), smoking(2), aging(2) and other 45(1) and 187(2) traits |
| cg19854704 | + | 10 | 95kb down *ENSG00000286295* | Long intergenic non-protein coding RNA 1164 | - | - | - |
| cg07728793 | - | 2 | *RAMP1* | Body | - | adolescent idiopathic scoliosis, leukocyte count, eosinophil count, intraocular pressure measurement, coronary artery calcification, type II diabetes mellitus | aging (1)(2), mortality (1)(2), obesity(1)(2), Triglycerides to total lipids ratio in medium HDL(2), Free cholesterol to total lipids ratio in large HDL(2), smoking(2) and other 19(1) and 59(2) traits |
| cg18072629 | - | 10 | *GATA3* | Intron | - | [asthma](https://www.ebi.ac.uk/gwas/search?query=asthma), [allergy](https://www.ebi.ac.uk/gwas/search?query=allergy), [Glucocorticoid use measurement](https://www.ebi.ac.uk/gwas/search?query=Glucocorticoid%20use%20measurement), [eosinophil count](https://www.ebi.ac.uk/gwas/search?query=eosinophil%20count), [colorectal cancer](https://www.ebi.ac.uk/gwas/search?query=colorectal%20cancer), [basal cell carcinoma](https://www.ebi.ac.uk/gwas/search?query=basal%20cell%20carcinoma), [hair shape measurement](https://www.ebi.ac.uk/gwas/search?query=hair%20shape%20measurement), [B-cell acute lymphoblastic leukemia](https://www.ebi.ac.uk/gwas/search?query=B-cell%20acute%20lymphoblastic%20leukemia),  [Hodgkin lymphoma](https://www.ebi.ac.uk/gwas/search?query=nodular%20sclerosis%20Hodgkin%20lymphoma), [fasting blood glucose measurement,](https://www.ebi.ac.uk/gwas/search?query=fasting%20blood%20glucose%20measurement)[HOMA-B,](https://www.ebi.ac.uk/gwas/search?query=HOMA-B)[fasting blood insulin measurement](https://www.ebi.ac.uk/gwas/search?query=fasting%20blood%20insulin%20measurement), [smoking behavior](https://www.ebi.ac.uk/gwas/search?query=smoking%20behavior), [thyroid stimulating hormone measurement](https://www.ebi.ac.uk/gwas/search?query=thyroid%20stimulating%20hormone%20measurement), [risk-taking behaviour](https://www.ebi.ac.uk/gwas/search?query=risk-taking%20behaviour), [rheumatoid arthritis](https://www.ebi.ac.uk/gwas/search?query=rheumatoid%20arthritis), [type I diabetes mellitus](https://www.ebi.ac.uk/gwas/search?query=type%20I%20diabetes%20mellitus), diet measurement, [influenza A (H1N1)](https://www.ebi.ac.uk/gwas/search?query=influenza%20A%20(H1N1)), [selective IgA deficiency disease](https://www.ebi.ac.uk/gwas/search?query=selective%20IgA%20deficiency%20disease) , [respiratory system disease](https://www.ebi.ac.uk/gwas/search?query=respiratory%20system%20disease), [systemic lupus erythematosus](https://www.ebi.ac.uk/gwas/search?query=systemic%20lupus%20erythematosus), [recalcitrant atopic dermatitis](https://www.ebi.ac.uk/gwas/search?query=recalcitrant%20atopic%20dermatitis), [stroke](https://www.ebi.ac.uk/gwas/search?query=stroke), [Nasal Cavity Polyp](https://www.ebi.ac.uk/gwas/search?query=Nasal%20Cavity%20Polyp), [Malignant epithelial tumor of ovary,](https://www.ebi.ac.uk/gwas/search?query=Malignant%20epithelial%20tumor%20of%20ovary)[response to paclitaxel](https://www.ebi.ac.uk/gwas/search?query=response%20to%20paclitaxel), [antiphospholipid syndrome](https://www.ebi.ac.uk/gwas/search?query=antiphospholipid%20syndrome), [Takayasu arteritis](https://www.ebi.ac.uk/gwas/search?query=Takayasu%20arteritis), [ankle injury](https://www.ebi.ac.uk/gwas/search?query=ankle%20injury) | smoking(2), aging(2), colorectal cancer (1), bariatric surgery (1) and other 40(1) and 60(2) traits |
| cg05005073 | + | 1 | 10kb up *NDUFS5* | Intergenic region | - | - | aging (1)(2), smoking (1)(2) and other 2(1) and 25(2) traits |
| cg09516627 | + | 2 | *CENPO* | TSS200 | - | BMI, body fat distribution, body height, sex interaction measurement | gestational diabetes mellitus (1), gestational age (1), smoking(1) and other 2(1) and 1(2) traits |
| **cg02727104** | + | 13 | 13kb down *SOHLH2* | Intergenic region | childhood acute lymphoblastic leukemia+  colorectal tumor +  child abuse+ | - | smoking associated colorectal carcinogenesis(1) and other 9(1) and 15(2) traits |
| cg26725813 | - | 15 | *ZNF770* | 5UTR | - | cup-to-disc ratio measurement, smoking | associated with 5 traits on (1) and 17 on (2) |
|  |  |  |  |  |  |  |  |
|  |  |  |  |  |  |  |  |
| cg05931551 | - | 1 | *TUT4* | Body | - | bodyheight,unipolar depression, joint hypermobility measurement, intelligence | smoking(1)(2), gestational diabetes mellitus(1), obesity(1), aging(1)(2), maternal smoking (1), bariatric surgery (1) and other 12(1) and 18(2) traits |
| cg26392737 | + | 8 | *DMTN* | 1stExon | sclerosimultipla -, aging - | total cholesterol measurement, low density lipoprotein cholesterol measurement | aging(1), smoking(1)(2), gestational age(1), maternal smoking(1), obesity (1) and other 30(1) traits |
|  |  |  |  |  |  |  |  |
|  |  |  |  |  |  |  |  |
| cg04512603 | - | 7 | *ZNF273* | Intron | - | Alzheimer's disease, age at onset | colorectal laterally spreading tumor(1), smoking(1), aging(2) and other 20(1) and 11(2) traits |
| cg23218354 | + | 1 | 50kb down *ACTRT2* | Intergenic region | Coffin–Siris syndrome +  SETD1B-related syndrome - | - | colorectal laterally spreading tumor(1), gestational diabetes mellitus(1), gestational age(1)(2), aging and other 7(1) and 15(2) traits |
|  |  |  |  |  |  |  |  |
| cg09855212 | + | 11 | *SYTL2* | 5UTR | - | body height, family history of Alzheimer's disease, waist-hip ratio, response to non-steroidal anti-inflammatory, drug-induced liver injury | smoking (1), aging(2), gestational age(2), maternal smoking(1)(2), Total cholesterol to total lipids ratio in large VLDL(2), Triglycerides to total lipids ratio in large VLDL(2) and other 16(1) and 37(2) traits |
| cg04305601 | - | 16 | *ZNF423* | Body | - | waist-hip ratio, BMI-adjusted waist-hip ratio, body height, smoking status measurement, heel bone mineral density | birthweight(2), aging(1)(2), obesity(1), smoking(1)(2), bariatric surgery(1), type 2 diabetes(1), colorectally spreading tumor(1), childhood obesity(1), colorectal cancer(1), maternal smoking(1), BMI(1), insulin resistance(1), Triglycerides to total lipids ratio in medium HDL (2),gestational age(2) and other 33(1) and 155(2) traits |
| cg13590166 | - | 10 | 70kb down *WAC* | long intergenic non-protein coding RNA 2652 | air pollution + | - | bariatric surgery(1), insulin resistance(1), mortality(1)(2), gestational age(1)(2) and other 9(1) and 29(2) traits |
| cg01807862 | - | 5 | *MCIDAS* | Body | B Acute Lymphoblastic Leukemia with t(12;21)(p13.2;q22.1); ETV6-RUNX1; +  gender + | - | colorectal cancer(1), colorectal laterally spreading tumor(1), aging(1),insuline resistance(1), smoing(1) and other 22(1) traits |
| cg14781041 | + | 1 | *SIPA1L2* | 1stExon | - | adolescent idiopathic scoliosis, cardiovascular disease, Parkinson's disease, systolic blood pressure | smoking(1), obesity(1), type 2 diabetes(1), aging(1)(2), colorectal cancer(1), gestational age(2), mortality(1)(2) and other 11(1) and 31(2) traits |
| cg15915658 | + | 11 | 30kb down *SCYL1* | Intergenic region | exercise + alcohol consumption  -- | - | smoking(1)(2), maternal smoking(1)(2), gestational age(2), gestational diabetes mellitus(1) and other 6(1) and 17(1) traits |
| cg03466415 | + | 2 | *SCN9A* | 5UTR | - | triacylglycerol 58:10 measurement, cannabis dependence measurement, schizophrenia, dental caries | colorectal cancer(1), aging(1)(2), obesity(1), smoking(1)(2), gestational age(1)(2) and other 8(1) and 24(1) traits |
| cg22453818 | + | 5 | *LHFPL2* | 5UTR | smoking - | platelet crit, platelet count, macula measurement, platelet component distribution width, selenium measurement | smoking(1)(2), aging(1)(2), BMI(1), obesity(1), gestational diabetes mellitus(1), type 2 diabetes(1), colorectal cancer(1), waist circumference(1)(2), mortality(1)(2), birthweight(2), gestational age(1)(2) and other 25(1) and 70(2) traits |
| cg07175848 | - | 16 | *SNTB2* | Body | - | blood metabolite measurement, high density lipoprotein cholesterol measurement, self reported educational attainment, body mass index, cognitive function measurement, heel bone mineral density, risk-taking behaviour, glomerular filtration rate, intelligence, memory performance, attention deficit hyperactivity disorder | smoking(1),aging(1)(2) and other 7(1) and 20(2) traits |
| cg18971416 | + | 10 | *THNSL1* | TSS1500 | - | response to diisocyanate, asthma | gestational diabetes mellitus(1) and other 7(1) and 3(2) traits |
|  |  |  |  |  |  |  |  |
| cg18152712 | + | 6 | *E2F3* | TSS1500 | - | body mass index, total cholesterol measurement, blood molybdenum measurement,physical activity | gestational age(1)(2), bariatric surgery(1) and other 18(1) and 29(2) traits |
| cg08060902 | - | 19 | ZNF709 | TSS1500 | bladder cancer - | - | aging(1)(2), gestational age(2), mortality(1)(2), gestational diabetes mellitus(1), smoking(1), colorectal laterally spreading tumor(1) and other 19(1) and 39(2) traits |
| cg04963607 | - | 7 | *PRKAR1B* | Body | - | erythrocyte count,  hematocrit, body height, hemoglobin measurement, Alopecia, Varicose veins, histidine measurement, amino acid measurement, tryptophan measurement, amino acid measurement, atrial fibrillation, body mass index, wellbeing measurement | birthweight(2), smoking(1), aging(1)(2), colorectal laterally spreading tumor(1), colorectal cancer(1), obesity(1),bmi(2) gestational age(1)(2), mortality(1)(2), gestational diabetes mellitus(1), type 2 diabetes(1), bariatric surgery(1), maternal smoking(1) and other 58(1) and 302(2) traits |
| cg00483825 | - | 10 | *CFAP46* | Body | - | gestational age, birth measurement, sclerosing cholangitis | colorectal laterally pread tumor(1), smoking(1), gestational diabetes mellitus(1), colorectal cancer(1), type 2 diabetes(1), aging(1), mortality(1), obesity(1), maternal smoking(1),gestational age(1),insulin resistance(1), BMI(1) and other 42(1) traits |
| cg18417562 | - | 5 | *CTNNA1* | Body | - | acute myeloid, leukemia, risk-taking behaviour, mean corpuscular, hemoglobin, schizophrenia, age-related hearing impairment | smoking(1)(2), type 2 diabetes(1),aging(1), obesity(1), colorectal cancer(1),waist circumference(1),gestational age(1)(2), colorectal laterally spreading tumor(1) and other 19(1) and 26(2) traits |
| cg09361653 | - | 17 | *RPTOR* | Body | - | body mass index, pulse pressure measurement, visceral adipose tissue measurement | smoking(1), maternal smoking(1),obesity(1), aging(1),colorectal cancer(1),type 2 diabetes(1),BMI(1),mortality(1),gestational diabetes mellitus(1), bariatric surgery(1), insulin resistance(1), colorectal laterally spread tumor(1), birth weight(1), gestational age(1) and other 89(1) traits |
| cg22896429 | + | 6 | *MTHFD1L* | Body | - | PHF-tau measurement, Alzheimer's disease, coronary heart disease, traffic air pollution measurement, peripheral arterial disease, schizophrenia | insulin(2), smoking(1)(2),aging(1)(2), gestational age(2), childhood obesity(1), bariatric surgery(1) and other 15(1) and 79(2) traits |
| cg19794939 | + | 11 | *LGR4* | TSS200 | - | balding measurement, heel bone mineral density, BMI, obese BMI status, urate measurement, waist-hip ratio, hair color, Alopecia, vital capacity, body weight, preterm premature rupture of the membranes, spontaneous preterm birth | smoking(1), birthweight(2), gestational age(2) and other 18(1) and 34(2) traits |
| cg03113121 | + | 19 | *FOXA3* | TSS1500 | - | FEV/FEC ratio, coronary artery disease, respiratory system disease, chronic obstructive pulmonary disease, coronary artery disease, waist-hip ratio, neuroticism measurement, red blood cell distribution width, subcutaneous adipose tissue measurement | smoking(1), aging(1), maternal bmi(2) and other 6(1) and 7(2) traits |
| cg23114964 | - | 1 | *HIVEP3* | 5UTR | Behcet's disease - | - | birthweight(2), smoking(1)(2), aging(1)(2), obesity(1), maternal obesity(2), maternal bmi(2), colorectal cancer(1), gestational diabetes mellitus(1), bariatric surgery(1), gestational age(1)(2), colorectal laterally spread tumor(1), type 2 diabetes(1)(2), Serum low-density lipoprotein cholesterol(2), mortality(2) and other 42(1) and 175(2) traits |
| cg06527318 | - | 1 | *MIR4425* |  | papillary thyroid carcinoma -  recurrent miscarriage - | - | smoking(1), maternal smoking(1), cardiovascular risk(1), colorectal cancer(1) and other 10(1) traits |
| cg20100049 | - | 11 | *KMT5B* | 5UTR | SETD1B-related syndrome aging - | heel bone mineral density, urate measurement, mean corpuscular hemoglobin, systolic blood pressure, serum IgG glycosylation measurement | birthweight(2), maternal smoking(1), aging(1)(2), gestational age(2), bariatric surgery(1) and other 12(1) and 35(2) traits |
|  |  |  |  |  |  |  |  |
| cg20515787 | + | 11 | SART1 | TSS1500 | bariatric surgery NR | - | bariatric surgery(1). smoking(1), maternal BMI(2), and other 6(1) and 34(2) traits |
| cg13680864 | + | 10 | STAM | TSS200 | - | aspartate aminotransferase measurement, allergy | mortality(1), gestational age(1)(2), and other 5(1) and 21(2) traits |
| cg23096644 | + | 7 | SGCE | Body | - | breast carcinoma, schizophrenia, neurofibrillary tangles measurement | aging(1), smoking(1)(2), gestational age(2), colorectal laterally spread tumor(1), obesity(1), type 2 diabetes(1), colorectal cancer(1) and other 31(1) and 18(2) traits |
| cg03722643 | - | 2 | *GPR45* | 1stExon | - | age at menarche, smoking behavior, unipolar depression, cervical carcinoma, type II diabetes mellitus | smoking(1), insulin resistance(1), maternal overweight/obesity(2), maternal BMI(2), mortality(1) and other 8(1) and 18(2) traits |
| cg19857227 | - | 14 | *ENSG00000240914* | Body | aging + | - | aging(1), colorectal cancer(1), colorectal laterally spread tumor(1), smoking(1), bariatric surgery(1) and other 12(1) traits |
| cg08707988 | - | 6 | *41 kb up LOC100294145* | Integenic region | - | - | - |
| cg09749788 | + | 8 | *MCPH1-AS1* | Body | osteoarthritis (OA) -  prenatal arsenic exposure + | - | aging(1), bariatric surgery(1), waist circumference(1) and other 9(1) traits |
| cg06655187 | + | 17 | *ZNHIT3* | Body | 17q12 deletion - | - | bariatric surgery(1), gestational age(2) and other 6(1) and 21(2) traits |
| cg10800369 | + | 12 | *LMO3* | TSS1500 | infertility - | - | aging(1)(2), gestational age(2), colorectal cancer(1), maternal smoking(1)(2), type 2 diabetes mellitus(1), mortality(1)(2) and other 18(1) and 63(2) traits |
| cg24864887 | + | 15 | *800b down ISL2* | Intergenic region |  |  | aging(1)(2), bariatric surgery(1), smoking(1) (2), gestational age(2) and other 14(1) and 49(2) traits |

S2Table: Traits associated in EWAS Atlas with saliva44-SGA-related CpGs. The second column reports the methylation status of each locus in our analysis. In the sixth column are reported the traits found to be associated with each locus in the EWAS Atlas and the methylation status for each CpG in the EWAS Atlas studies (+=hypermethylated; -=hypomethylated; NR=not reported). In the 7th column are reported the traits found to be associated with genetic variants of the related nearest genes in the GWAS Catalog. In the last column are reported traits associated with DNA methylation at gene level.

| **CpGs** | **methylation**  **status** | **CHR** | **Gene on which CpG is allocated or nearest gene** | **CpG location on the gene** | **Traits associated with DNA methylationin EWAS Atlas** | **Traits associated with gene variantsin GWAS catalog** | **Traits associated with DNA methylation at gene level in EWAS Atlas (1) and EWAS Catalog (2)** |
| --- | --- | --- | --- | --- | --- | --- | --- |
| cg26168577 | + | 17 | *MRC2* | Body | - | pulse pressure measurement,  blood protein measurement,  heel bone mineral density,  FEV/FEC ratio,  systolic blood pressure,  diastolic blood pressure,  vital capacity,  mathematical ability,  wellbeing measurement,  neuroticism measurement,  depressive symptom measurement, intelligence | maternal overweight/obesity(2), aging(1)(2), gestational age(2), colorectal cancer(1), colorectal laterally spread tumor (1), smoking(1)(2), gestational diabetes mellitus(1) and other 20(1) and 63(2) traits |
| cg03066788 | - | 8 | *PNOC* | 5UTR | obesity -  facial aging + | [body mass index](https://www.ebi.ac.uk/gwas/search?query=body%20mass%20index), [serum IgG glycosylation measurement](https://www.ebi.ac.uk/gwas/search?query=serum%20IgG%20glycosylation%20measurement) | aging(1)(2), gestational age(2), type 2 diabetes(1), colorectal laterally spread tumor(1), obesity(1), and other 12(1) and 26(2) traits |
| cg26615232 | - | 1 | *USH2A* | Body | - | smoking status measurement, DNA methylation, cognitive function measurement, self reported educational attainment, birthweight, unipolar depression, alcohol dependence, helping behavior measurement, chronic mucus hypersecretion, serum IgG glycosylation measurement, testosterone measurement, eye color measurement,skin pigmentation measurement | gestational age(2), colorectal cancer(1), colorectal laterally spread tumor(1), aging(1)(2), smoking(1)(2), mortality(1)(2) and other 14(1) and 31(2) traits |
| cg12055114 | + | 19 | *LMNB2* | TSS1500 | - | erythrocyte count, pulse pressure measurement, hematocrit hemoglobin measurement, waist-hip ratio, self reported educational attainment, Hodgkins lymphoma, risk-taking behaviour, cleft palate, cleft lip, body height, insulin metabolic clearance rate measurement, glucose homeostasis measurement, wellbeing measurement, sodium measurement, diastolic blood pressure,  BMI-adjusted hip circumference | aging(1)(2), bariatric surgery(1), maternal overweight/obesity(2), BMI(1),maternal BMI(2),gestational age(2), smoking(1), obesity(1) and other 14(1) and 39(2) traits |
| cg04873627 | + | 4 | *TBC1D19* | Intron |  | Varicose veins  acute myeloid leukemia  diastolic blood pressure  systolic blood pressure  cardiovascular disease  Agents acting on the renin-angiotensin system use measurement  schizophrenia  reaction time measurement  superior frontal gyrus grey matter volume measurement | aging(1)(2), gestational age(2) and other 8(1) and 15(2) traits |
| cg15225594 | + | 5 | 20kb down *ERCC8* | Intergenic region | - | - | aging(2) and other 10(1) and 4(2) traits |
| cg01286950 | - | 2 | *AFF3* | Body | - | self reported educational attainment, mathematical ability, erythrocyte count, body mass index, rheumatoid arthritis, serum non-albumin protein measurement, intelligence, self reported educational attainment, BMI-adjusted waist-hip ratio, schizophrenia, intelligence, self reported educational attainment, cognitive function measurement, smoking status measurement, waist-hip ratio, albumin:globulin ratio measurement, balding measurement, rheumatoid arthritis, celiac disease, mean corpuscular hemoglobin, social interaction measurement, blood protein measurement, platelet crit, gastroesophageal reflux disease, acute myeloid leukemia, intraocular pressure measurement, vital capacity,age at first birth measurement, body height, serum IgG measurement, grip strength measurement, type 1 diabetes nephropathy, household income, age at menarche, FEV/FEC ratio, type I diabetes mellitus, alcohol consumption measurement, adolescent idiopathic scoliosis, type 1 diabetes nephropathy, chronic kidney disease, systemic juvenile idiopathic arthritis, polyarticular juvenile idiopathic arthritis, rheumatoid factor negative, oligoarticular juvenile idiopathic arthritis, cannabis dependence measurement, number of children ever born measurement, schizophrenia, response to paliperidone, schizophrenia symptom severity measurement, Astigmatism, susceptibility to chronic sinus infection measurement, mammographic density measurement | colorectal cancer(1), maternal smoking(1)(2), mortality(1), gestational age(2). aging(1)(2), type 2 diabetes(1), colorectal laterally spread tumor(1), maternal overweight/obesity(2), maternal BMI(2), BMI(1), amount of visceral adipose tissue(1) and other 38(1) and 118(2) traits |
| cg18072629 | + | 10 | *GATA3-AS1* | Intron |  | adult onset asthma, Glucocorticoid use measurement, B-cell acute lymphoblastic leukemia, childhood onset asthma, nodular sclerosis Hodgkin lymphoma, Inhalant adrenergic use measurement, allergy, acute lymphoblastic leukemia, Hodgkins lymphoma, asthma, type I diabetes mellitus, balding measurement, rheumatoid arthritis, selective IgA deficiency disease, respiratory system disease, susceptibility to childhood ear infection | - |
|  |  |  |  |  |  |  |  |
|  |  |  |  |  |  |  |  |
| cg13984701 | + | 9 | *COL5A1* | Body | - | central corneal thickness, corneal topography, intraocular pressure measurement, eye measurement, waist-hip ratio, phosphatidylcholine measurement, mean arterial pressure, longevity, response to bronchodilator, FEV/FEC ratio, Graves disease, Thyrotoxic periodic paralysis, Crohn's disease | smoking(1)(2), gestational age(2), aging(1)(2), colorectal laerally spreading tumor(1), mortality(1)(2), gestational diabetes mellitus(1), maternal smoking(1), birthweight(1), colorectal cancer(1), obesity(1), maternal overweight/obesity(2), maternal BMI(2), and other 27 traits |
| cg26332310 | - | 1 | *BARHL2* | 1stExon | aging (NR)  acute lymphoblastic leukemia +[  colorectal cancer + | - | aging(1), colorectal laterally spread tumor(1), bariatric surgery(1), colorectal cancer(1) and other 13(1) and 133(2) traits |
|  |  |  |  |  |  |  |  |
| cg24391471 | - | 3 | *ZDHHC3* | Body | prenatal arsenic exposure + | macrophage inflammatory protein 1b measurement, t-tau:beta-amyloid 1-42 ratio measurement, bipolar disorder, depressive symptom measurement, response to antidepressant, acne | smoking(1)(2), obesity(1), bariatric surgery(1) and other 10(1) and 7(2) traits |
| cg02547025 | - | 2 | *LBH* | TSS200 | aging +  maternal artritereumatoide +  bariatric surgery (NR) | - | bariatric surgery(1), smoking(1)(2), type 2 diabetes(1), aging(1)(2), obesity(1), gestational age(1)(2), colorectal cancer(1) and other 17(1) and 31(2) traits |
| cg23508813 | + | 3 | 2.5kb down *LOC100507274* | Intergenic region | acute lymphoblastic leukemia + | - | - |
| cg26589351 | - | 17 | *SPOP* | 5UTR | - | mean corpuscular volume, mean corpuscular hemoglobin, asthma, basophil count, eosinophil count, FEV/FEC ratio, prostate carcinoma, adult onset asthma, heel bone mineral density, cancer, milk allergy measurement | aging(1)(2), smoking(1)(2), maternal smoking(2), gestational age(2), birthweight(1)(2) and other 5(1) and 27(2) traits |
| cg06234201 | + | 16 | *DUS2* | Intron | down syndrome +  mortality - | - | smoking(1), bariatric surgery(1), aging(1), type 2 diabetes(1), gestational diabetes mellitus(1), mortality(1), gestational age(1), BMI(1) and other 24(1) traits |
|  |  |  |  |  |  |  |  |
|  |  |  |  |  |  |  |  |
|  |  |  |  |  |  |  |  |
|  |  |  |  |  |  |  |  |
| cg23954819 | - | 12 | *BRI3BP* | TSS200 | maternal Hepatitis B virus + | - | gestational diabetes mellitus(1) and other 6(1) and 20(2) traits |
| cg19445996 | - | 17 | *RAB37* | TSS200 | - | blood protein measurement, leukocyte count, monocyte count, C-reactive protein measurement, red blood cell distribution width, myeloid white cell count, fibrinogen measurement, monocyte percentage of leukocytes, neutrophil count, eosinophil count, granulocyte count, platelet crit, neutrophil count, basophil count, lymphocyte count, neutrophil count, coronary artery disease, non-alcoholic fatty liver disease, Hepatic fibrosis, breast carcinoma | aging(1)(2), gestational age(2), smoking(1)(2), colorectal laterally spreading tumor(1), obesity(1), waist circumference(2), High-density lipoprotein cholesterol(2) and other 34(1) and 59(2) traits |
| cg02980127 | + | 14 | 14,5kb down *ENSG00000258736.1* | Intergenic region | - | - | smoking(1), gestational age(2) and other 6(1) and 14(2) traits |
| cg22869025 | + | 20 | *ACSS1* | Body | - | cystatin-F measurement, blood protein measurement, alcohol consumption measurement, alkaline phosphatase measurement, bitter alcoholic beverage consumption measurement, volumetric bone mineral density, neuropsychological test | aging(1)(2), gestational age(3), obesity(1) and other 9(1) and 32(2) traits |
| cg19403534 | + | 10 | *PRLHR* | TSS1500 | - | pancreatic carcinoma, exercise, body height, empathy measurement | aging(1)(2), gestational diabetes mellitus(1), adiposity(1), obesity(1) and other 10(1) and 34(2) traits |
| cg12322146 | + | 3 | 125kb down *RBMS3* | Intergenic region | colorectal laterally spreading tumor - | - | aging(1)(2), colorectal cancer(1), colorectal laterally spreading tumor(1), BMI(1), obesity(1), gestational diabetes mellitus(1), type 2 diabetes(1), gestational age(1)(2), smoking(1) and other 29(1) and 49(2) traits |
| cg06458489 | - | 5 | 150kb down *SNORA63* | Intergenic | - | - | associated with 1 traits in (1) |
| cg00383136 | + | 6 | *HLA-DRA* | Body | gestational diabetes mellitus + | - | aging(1)(2), gestational age(1)(2), maternal overweight/obesity(2), maternal BMI(2), smoking(1)(2) and other 18(1) and 49(2) traits |
| cg16899265 | - | 18 | *GRP* | TSS1500 | down syndrome - | - | birthweight(2), aging(1), gestational diabetes mellitus(1), obesity(1), colorectal laterally spreading tumor(1) and other 10(1) and 22(2) traits |
| cg15847996 | + | 10 | *INPP5A* | Body | - | heel bone mineral density, systolic blood pressure, pulse pressure measurement, cardiovascular disease, sclerosing cholangitis, migraine disorder, triglyceride measurement, body height, gestational age, birth measurement, gait measurement, interleukin 9 measurement, smoking behavior, 3-hydroxy-1-methylpropylmercapturic acid measurement, periprostheticosteolysis, total hip arthroplasty, disease progression measurement, atypical femoral fracture, response to bisphosphonate, periodontitis, smoking behavior, 3-hydroxypropylmercapturic acid measurement | smoking(1), gestational diabetes mellitus(1), obesity(1),maternal overweight/obesity(2),maternal BMI(2), aging(1)(2), type 2 diabetes(1), mortality(1), maternal smoking(1)(2), colorectal laterally spreading tumor(1), fat mass index(1), waist circumference(1)(2), colorectal cancer(1), gestational age(1)(2), BMI(1), insuline resistance(1) and other 73(1) and 494(2) traits |
| cg08715720 | + | 5 | *PPP2R2B* | Body | - | chronotype measurement, mathematical ability, schizophrenia, attention deficit hyperactivity disorder, unipolar depression, schizophrenia, autism spectrum disorder, bipolar disorder, alcohol dependence, neuropsychological test, urate measurement, underweight body mass index status, anxiety disorder, cognitive behavioural therapy, reaction time measurement, sleep quality, susceptibility to chickenpox measurement | smoking(1)(2), aging(1)(2), colorectal cancer(1), colorectal lateral spreading tumor(1), obesity(1), type 2 diabetes(1), bariatric surgery(1), gestational age(1)(2), waist circumference(1), Triglycerides to total lipids ratio in very large HDL(2) and other 20(1) and 65(2) traits |
| cg20388707 | + | 2 | *NGEF* | Body | amount of visceral adipose tissue - | - | obesity(1), type 2 diabetes(1), aging(1)(2), smoking(1)(2), maternal smoking(1), amount of visceral adipose tissue(1), maternal BMI(2),gestational age(2) and other 24(1) and 95(2) traits |
| cg01504555 | - | 16 | *SYCE1L* | 3'UTR | - | [beta-amyloid 1-42 measurement](https://www.ebi.ac.uk/gwas/search?query=beta-amyloid%201-42%20measurement), susceptibility to childhood ear infection measurement, type II diabetes mellitus | maternal BMI(2), gestational age(2) and other 8(1) and 9(2) traits |
| cg07965823 | - | 14 | *ISM2* | Body | aging + | - | bariatric surgery(1), aging(1), gestational age(2), BMI(1) and other 6(1) and 26(2) traits |
| cg20244295 | + | 15 | *ZNF710* | 3'UTR | - | body height, type II diabetes mellitus | fasting insulin(2), smoking(1)(2), maternal smoking(2), aging(1)(2), gestational age(1)(2), maternal smoking(1), BMI(1)(2), waist circumference(2), obesity(1), colorectal cancer(1), gestational diabetes mellitus(1) and other 37 traits |
| cg09311778 | - | 17 | 2kb down *KAT7* | Intergenic region | - | - | aging(1), insulin resistance(1) and other 12(1) and 75(2) traits |
| cg05754929 | - | 16 | *GSE1* | Intron | - | platelet component distribution width  eosinophil count  balding measurement  body height  platelet count  systolic blood pressure  platelet crit  leukocyte count  waist-hip ratio  BMI-adjusted waist-hip ratio  Alopecia  triglyceride measurement  drug-induced agranulocytosis, response to sulfasalazine  blood cobalt measurement  alopecia areata  sporadic amyotrophic lateral sclerosis  pulse pressure measurement, diastolic blood pressure, systolic blood pressure, hypertension | aging(1), smoking(1), obesity(1), maternal smoking(1), gestational age(1), type 2 diabetes(1), BMI(1), mortality(1), insulin resistance(1), bariatric surgery(1), colorectal laterally spreading tumor(1), colorectal cancer(1), gestational diabetes mellitus(1) and other 95(1) traits |
| cg18246134 | - | 3 | *LINC02877* | TSS1500 | - | response to vaccine, cytokine measurement, cerebral amyloid deposition measurement, sporadic amyotrophic lateral sclerosis | maternal overweight/obesity(2) and other 4(1) and 7(2) traits |
| cg21688288 | + | 21 | *PDE9A* | Body | - | high density lipoprotein cholesterol measurement, metabolic syndrome, FEV/FEC ratio | birthweight(2), maternal BMI(2), maternal overweight/obesity(2), colorectal cancer(1), smoking(2), gestational age(2), aging(1)(2) and other 15(1) and 55(2) traits |
| cg22159939 | - | 1 | 30kb down *CENPF* | Intergenic region | - | - | birthweight(2), mortality(2), aging(1)(2), obesity(1), bariatric surgery(1), maternal smoking(1)(2), smoking(1)(2), colorectal laterally spreading tumor(1) and other 10(1) and 35(2) traits |
| cg25271404 | - | 15 | *GOLGA8B* | Body | - | FEV change measurement, response to bronchodilator, chronic obstructive pulmonary disease | aging(1), bariatric surgery(1), smoking(2), gestational age(2) and other 2(1) and 14(2) traits |
| cg24745895 | + | 7 | 5kb up *AKR1D1* | Intergenic region | - | - | colorectal cancer(1), colorectal laterally spreading tumor(1), agin(1)(2), gestational age(2) and other 17(1) and 15(2) traits |
| cg17836487 | - | 2 | *TMEM131* | Body | Sjögren's syndrome. +  asthma +  alcohol consumption - | - | aging(1)(2), smoking(1)(2), gestational age(1)(2), type 2 diabetes(1)(2) and other 23(1) and 49(2) traits |
| cg24113784 | + | 17 | *MIEF2* | Body | - | - | bariatric surgery(1), smoking(1), aging(1) and other 3(1) traits |
| cg00701706 | - | 17 | *OTOP3* | TSS1500 | aging +  +  gestational age +  alcohol consumption - | - | aging(1)(2), gestational age(1)(2), maternal smoking(1)(2), colorectal cancer(1) and other 5(1) and 59(2) traits |
| cg24663455 | - | 10 | 50,5kb down *MGMT* | Intergenic region | - | - | birthweight(2), obesity(1),maternal overweight/obesity(2), maternal BMI(2), smoking(4), type 2 diabetes(1), smoking(1), aging(1)(2), gestational diabetes mellitus(1), mortality(1), gestational age(1)(2), mortality(2) and other 35(1) and 203(2) traits |
|  |  |  |  |  |  |  |  |
| cg08935613 | - | 11 | *HYLS1* | TSS1500 | - | estrogen-receptor negative breast cancer, survival time | smoking(1)(2), gestational age(2), bariatric surgery(1) and other 3(1) and 23(2) traits |
|  |  |  |  |  |  |  |  |
| cg22213242 | + | 11 | *CD248* | 1stExon | aging +  + | - | birthweight(1)(2), aging(1)(2), smoking(1)(2), maternal smoking(1)(2), obesity(1), gestational age(2) and other 15(1) and 29(2) traits |
| cg24420742 | + | 12 | *NAV3* | Intergenic region | alcol consumption - | - | aging(1), gestational age(2), smoking(1), colorectal cancer(1), colorectal laterally spreading tumor(1) and other 24(1) and 20(2) traits |
|  |  |  |  |  |  |  |  |

**References**

1. R Core Team. R: A language and environment for statistical computing. R Foundation for Statistical Computing, Vienna, Austria. (2017). <https://www.R-project.org/>.
2. Aryee MJ, Jaffe AE, Corrada-Bravo H, Ladd-Acosta C, Feinberg AP, Hansen KD, et al. Minfi: A flexible and comprehensive Bioconductor package for the analysis of Infinium DNA Methylation microarrays. *Bioinformatics*. 2014;**30**:1363-1369.
3. Touleimat N, Tost J. Complete pipeline for Infinium(®) Human Methylation 450K BeadChip data processing using subset quantile normalization for accurate DNA methylation estimation. *Epigenomics*. 2012;**4**:325-341.
4. Chen Y, Lemire M, Choufani S, Butcher DT, Grafodatskaya D, Zanke BW, et al. Discovery of cross-reactive probes and polymorphic CpGs in the IlluminaInfinium HumanMethylation450 microarray. *Epigenetics*. 2013;**8**:203-209.
